# Primary healthcare services’ accessibility and quality under compulsory social health insurance in Kazakhstan

**DOI:** 10.3389/fpubh.2024.1418367

**Published:** 2024-10-15

**Authors:** Makhabbat Shurenova, Kuralbay Kurakbayev, Tleukhan Abildaev, Aigul Tazhiyeva

**Affiliations:** ^1^Kazakhstan Medical University “Higher School of Public Health”, Almaty, Kazakhstan; ^2^Almaty Branch of the Non-Profit Joint Stock “Social Health Insurance Fund”, Almaty, Kazakhstan; ^3^S.D. Asfendiyarov Kazakh National Medical University, Almaty, Kazakhstan

**Keywords:** health insurance, healthcare financing, health expenditure, primary healthcare services, Kazakhstan

## Abstract

**Introduction:**

Between 2020 and 2022, there was a rise in employment and entrepreneurial activity, despite some unemployment growth. In the Compulsory Social Health Insurance (CSHI), insured individuals, especially privileged and wage workers, increased. However, certain contributors, like those exempt from contributions and single social taxpayers, decreased, possibly due to legislative changes or the economic climate. The study aimed to evaluate the effectiveness and accessibility of medical services within the frameworks of Compulsory Social Health Insurance and the state Guaranteed volume of free medical care based on data regarding waiting times and the volume of services provided.

**Methods:**

This retrospective study analyzed data from 2020 to 2022 on patients receiving care under Kazakhstan’s Mandatory Social Medical Insurance System (MSMIS) and Guaranteed Volume of Free Medical Care. Data included insurance status, labor market indicators, and medical service procurement. Descriptive statistics were calculated, and *t*-tests, with *p*-values indicating statistical significance (*p* < 0.05).

**Results:**

It has been identified that overall, the execution of the plan for all types of medical care in Kazakhstan, including Almaty, reaches 100.0%, indicating sufficient financial support for the healthcare system. Rehabilitation centers and COVID-19 testing services stand out with shorter waiting times, whereas comprehensive diagnostic and advisory services require significantly longer waiting periods. The Guaranteed Volume of Free Medical Care typically offers a greater number of services with shorter waiting times compared to MSMIS, except for specialized medical care services in diagnosing new formations, where the Guaranteed Volume of Free Medical Care also has shorter waiting times but provides a greater number of services.

**Conclusion:**

The study has allowed us to identify differences in the availability and volume of medical services provided between Compulsory Social Health Insurance and the state Guaranteed volume of free medical care.

## 1 Introduction

Over the past decade, most low- and middle-income countries have voiced support for Universal Health Coverage (UHC). Initially, strategies for universal health insurance focused on expanding access to the formal sector as a first step, but it is now recognized that coverage of the entire population, especially the poor, is necessary. However, assuming that mandatory schemes will automatically cover target population groups is mistaken. Little is known about the reasons why medical organizations may fail to comply with enrollment requirements, although a lack of knowledge and low quality of healthcare in public hospitals play a role (1, 2). In recent years, governments of rapidly aging countries have turned their attention to implementing state insurance schemes to help cover the expenses of caring for their older adult citizens (3).

Although being uninsured affects utilization, financial barriers persist for those with insurance who seek medical care. The strengths and weaknesses of mandatory health insurance systems provide valuable lessons for policymakers in low- and middle-income countries grappling with achieving equitable coverage through health insurance programs and the complex nature of financial barriers to access (4). Currently, many low- and middle-income countries striving for universal healthcare coverage face issues such as taxation versus social medical insurance; population coverage and benefits; single schemes versus multiple schemes; procurement and provider payment methods; and the role of policy and political commitment. However, there are also challenges associated with the dominance of private service providers paid on a fee-for-service basis, rapid population aging, and the combination of public and private sectors linked to private medical insurance (5). Expanding access to primary healthcare is associated with a lower rate of preventable hospitalizations for conditions related to outpatient care, confirming its use as an indicator of the quality of primary healthcare (6). A significant portion of people with physical disabilities report experiencing difficulties accessing adequate primary healthcare services (7, 8).

The results indicate that improving fairness in healthcare financing and access to medical services was a key motivation for merging medical insurance funds. Obstacles included resistance from groups with better benefits and concerns from workers and employers regarding increased contribution rates. The benefits of merging include enhancing fairness, reducing inequality in access and utilization of medical services, improving efficiency, and reducing administrative costs. The experiences of countries like South Korea, Turkey, Thailand, and Indonesia underscore the importance of political support and economic growth for the successful implementation of merged medical insurance funds (9). In the conducted study, respondents indicated that merging medical insurance funds has several advantages, such as controlling overall expenses, improving strategic procurement, and reducing administrative costs. However, some drawbacks include the reluctance of social protection agencies to collect insurance contributions, increased dissatisfaction among groups with generous benefits, the risk of financial fraud, and financial pressure on service providers in case of delayed payments (10).

The passage of the Affordable Care Act in 2010 led to significant improvements in coverage through subsidies for private insurance for the uninsured, the expansion of the Medicaid program (in some states), and enhanced protection for insured individuals. Funding for primary healthcare and public health also increased, with quality and expenditure being regulated through financial incentives for providing more efficient medical care (11).

The Alma-Ata Declaration and the Astana Declaration on Primary Health Care in 2018 emphasize the importance of primary healthcare for universal healthcare coverage and the Sustainable Development Goals agenda until 2030 (12).

Kazakhstan is undergoing a comprehensive healthcare reform aimed at restructuring its public healthcare system and improving primary healthcare services (13). Improving access to medications, interpersonal communication skills, and technical assistance are three main priorities for enhancing the perceived quality of primary healthcare and health policy measures (14).

In Kazakhstan, citizens receive free medical care covering preventive, diagnostic, and treatment services, as well as pharmaceutical assistance. This care, funded by national and local budgets, includes specialized, emergency, and inpatient care, along with medical rehabilitation. The guaranteed volume of free medical care includes primary and consultative-diagnostic services for socially significant and infectious diseases, emergency assistance, and forensic medical expertise.

The Social Health Insurance Fund, fully financed by the government, was established on July 1, 2016, by Resolution No. 389 of the Government of the Republic of Kazakhstan. Its creation and development were driven by the implementation of the CSHI system within the framework of the “Kazakhstan-2050” strategy, the “100 Concrete Steps” plan, and the law on social health insurance. The introduction of CSHI in 2020 was the result of the gradual development of the country’s healthcare system, which faced economic difficulties in the post-Soviet period.

In 1996, a mandatory health insurance system was introduced, but insufficient funding led to its cancellation in 1998. Subsequent implementation of mandatory insurance led to increased utilization of consultative-diagnostic services, especially within primary healthcare. In 2020, there were 2.8 consultative-diagnostic services per primary healthcare visit, in 2021–2024 services, and in 2022—already 6 services per visit to a primary care physician.

The intensification of efforts to ensure the technical quality of medical care itself does not necessarily lead to an improvement in clients’ perception of the quality of medical care and their willingness to use medical services in healthcare facilities (15). Authorized and practicing physicians should not overlook the presumed social contract and care models that form the basis of interaction with the service (16).

Due to the lack of clear distinction between guaranteed volumes and medical services provided to citizens on a paid basis, free medical care is being replaced by various paid medical services, particularly medical insurance (17).

Furthermore, there is a need to focus on studying effective methods of popularizing information about the basic health insurance system, supporting literacy in public policy, and improving the healthcare system environment to reduce catastrophic expenditures and eliminate financial barriers to access among certain groups (18, 19). High literacy in medical insurance is associated with widespread utilization of medical services, including primary care and preventive measures. Individuals with low literacy often delay or avoid seeking medical assistance due to a lack of understanding of their insurance policies and financial obligations (20). Furthermore, research is needed to study best practices for including vulnerable groups in medical insurance schemes (21).

The study aimed to evaluate the effectiveness and accessibility of medical services within the frameworks of Compulsory Social Health Insurance and the state Guaranteed Volume of Free Medical Care based on data regarding waiting times and the volume of services provided.

## 2 Materials and methods

### 2.1 Study design, population, and sampling technique

This retrospective study includes all patients receiving medical care within the Mandatory Social Medical Insurance System (MSMIS) and the Guaranteed Volume of Free Medical Care in outpatient and inpatient settings in Kazakhstan, including in the city of Almaty, for the years 2020–2022. Since the beginning of 2020, Kazakhstan has implemented a mandatory social medical insurance system. According to the Ministry of National Economy, out of 2,024,989 residents of the city of Almaty, the number of insured populations amounted to 81.1%. The number of uninsured individuals is 382,940 people (18.9%). The largest number of medical service providers is located in Almaty—190. For Kazakhstan we analyzed data on the main indicators of the labor market, the insurance status of Kazakh citizens by category of payers, the execution of the procurement plan by types of medical services, and to study the availability of medical services within the framework of mandatory social health insurance and the state guaranteed volume of free medical care we analyzed the waiting time for consultative and diagnostic services and types of services in primary health care in the city of Almaty.

### 2.2 Data collection

A retrospective study was conducted using data on citizens’ insurance status in the Republic of Kazakhstan, broken down by payer categories, labor market indicators, and procurement plan execution for various types of medical services from January 1, 2020, to December 31, 2022. The data were obtained from the Annual Report of the Social Health Insurance Fund for 2022. Payer categories were identified based on insurance status and procurement plan execution for different types of medical services in 2022. In 2022, the number of individuals entitled to receive medical care in the MSMIS system was 82.4% (*n* = 16,266,563), while the number of uninsured individuals was 17.6% (*n* = 3,474,720).

### 2.3 Ethical consideration

The study was conducted in accordance with the Declaration of Helsinki and approved by the Ethics committee of the Kazakhstan Medical University “KSPH” (IRB –15-2023 from 17 May 2023).

### 2.4 Statistical analysis

For analytical and descriptive analysis, data were obtained on the number of payer categories, their share of the total population, standard deviation (SD), *t*-test results, and *p*-value for each category. For each payer category, their number and percentage share of the total population for each of the 3 years were provided. The *t*-test and *p*-value were used to determine the statistical significance of differences between groups. The *p*-value (P) indicates the probability of obtaining the observed differences between groups given that the null hypothesis is true. If the *p*-value is less than the chosen significance level (usually 0.05), the differences are considered statistically significant. Statistical analysis of the results was conducted using SPSS version 25.0 (IBM, Armonk, New York, United States). A significant difference was observed between the years (*p* ≤ 0.001) (Table 1).

**Table 1:** Main indicators of the labor market in the Republic of Kazakhstan.

Category	2020	2021	2022
Labor force, thousand people	9180.8	9256.8	9429.8
Employed population, thousand people	8732.0	8807.1	8971.5
Hired employees, thousand people	6686.7	6710.2	6847.3
Self-employed, thousand people	2045.4	2096.9	2124.2
Unemployed population, thousand people	448.8	449.6	458.3
Unemployment rate, %	4.9	4.9	4.9
Youth unemployment rate, % (ages 15–24)^1^	3.8	3.7	3.8
Youth unemployment rate, % (ages 15–28)^2^	3.8	3.8	3.8
Long-term unemployment rate, %	2.2	2.1	2.3
Average duration of unemployment^3^, months	6.0	7.2	7.3
Persons not included in the labor force, 1,000 people^4^	4076.8	4093.3	4301.4

1 Age of classification as youth according to the standards of the International Labor Organization.

2 The age of classification as a youth according to the Law of the Republic of Kazakhstan “On State Youth Policy in the Republic of Kazakhstan”.

3 Excluding pensioners, full-time students, and disabled people classified as “unemployed”.

4 Hereinafter, the data is generated based on the results of a sample survey of 208,275 households (4.48% of the total number of households), the frequency of the survey is once a year.

## 3 Results

According to official data, the workforce in the country increased from 9180.8 thousand people in 2020 to 9429.8 thousand people in 2022. This may indicate an increase in the working-age population or changes in labor force participation. The number of employed individuals also increased from 8732.0 thousand people in 2020 to 8971.5 thousand people in 2022, indicating growth in job opportunities or increased labor force participation. The number of wage workers and self-employed individuals also increased over the same period, which may indicate growth in entrepreneurial activity and the creation of new jobs. Although the number of unemployed individuals increased from 448.8 thousand people in 2020 to 458.3 thousand people in 2022, the unemployment rate remained stable at 4.9%. This suggests that the increase in the workforce was accompanied by an increase in the number of employed individuals. The youth unemployment rate remained stable at 3.8% for both age groups (15–24 years and 15–28 years).

The long-term unemployment rate has slightly increased from 2.2% in 2020 to 2.3% in 2022. The average duration of unemployment has also slightly increased from 6.0 months in 2020 to 7.3 months in 2022, indicating difficulties in finding employment for some segments of the population. The number of individuals not in the labor force has also increased from 4076.8 thousand people in 2020 to 4301.4 thousand people in 2022. This could be attributed to various factors such as demographic changes or changes in labor force participation.

Based on this analysis, it can be concluded that there is a stable growth in the workforce and employment in the country while maintaining a relatively low unemployment rate, albeit with some increases in long-term unemployment and average duration of unemployment.

As of 2022, the mandatory social medical insurance system has registered 16.3 million individuals (Table 2), accounting for 82.4% of the total eligible population for medical assistance. The majority of these individuals belong to privileged citizen categories—11.4 million people (58.3%), as well as wage workers—4.9 million people (24.8%). Most categories of contributors have experienced a decrease in their share of the total population over the 3 years. However, there are exceptions, with the “Wage Workers” category experiencing an increase in its share. All changes in the shares of contributor categories to the total population are statistically significant, as the *p*-value (P) for each category is less than the chosen significance level of 0.05. Significant changes in the numbers of some contributor categories have been noted over the 3 years, with the “Wage Workers” category experiencing a sharp increase in 2021. Standard deviation values differ for different categories, indicating varying degrees of variability in the shares to the total population within each category. The category with the highest variability is “Payers of a single aggregate payment of the Unified Social Payment System” with an SD of 840291.4, while the category with the lowest variability is “Individuals working under civil contracts” with an SD of 21638.85.

**Table 2:** Data on the insurance status of citizens of the Republic of Kazakhstan by payer category.

Payer categories	2020		2021		2022		SD	*T*-test	*P*
	Population	Share of the total number, in %	Population	Share of the total number, in %	Population	Share of the total number, in %			
Persons for whom deductions and (or) contributions were paid	17,424,525	92.30 ± 0.01	17,467,568	91.30 ± 6.4	17,126,231	86.70 ± 0.01	185895.6	396.0	*p* ≤ 0.001
Persons exempt from paying contributions	11,182,907	59.20 ± 0.01	11,358,652	59.40 ± 0.01	11,481,325	58.30 ± 0.01	149993.5	63.6	*p* ≤ 0.001
Wage-earners	4,245,642	22.50 ± 0.01	5,372,893	28.10 ± 0.01	4,896,038	24.80 ± 0.01	565847.5	162.6	*p* ≤ 0.001
Individual entrepreneurs and persons engaged in private practice	231,034	1.20 ± 0.003	289,443	1.50 ± 0.00	316,848	1.60 ± 0.003	43830.52	94.3	*p* ≤ 0.001
Individuals working under civil contracts	85,263	0.50 ± 0.002	125,938	0.70 ± 0.00	118,402	0.50 ± 0.002	21638.85	0.00	*p* ≥ 0.05
Payers of a single aggregate payment of the Unified Social Payment System	1,655,405	8.80 ± 0.007	199,088	1.00 ± 0.00	200,869	1.00 ± 0.002	840291.4	1071.4	*p* ≤ 0.001
Self-payers	24,274	0.10 ± 0.0007	121,554	0.60 ± 0.00	112,749	0.50 ± 0.002	53803.27	188.7	*p* ≤ 0.001
Persons entitled to receive medical care in the compulsory medical insurance system	15,845,537	83.90 ± 0.008	15,527,249	81.30 ± 0.01	16,266,563	82.40 ± 0.01	370844.8	117.1	*p* ≤ 0.001
Uninsured	3,031,591	16.10 ± 0.008	3,598,371	18.70 ± 0.01	3,474,720	17.60 ± 0.01	298019.1	117.1	*p* ≤ 0.001
Total population	18,877,128	100.0	19,102,465	100	19,741,283	100	448261.3		

Based on the conducted analysis, it can be concluded that there are statistically significant changes in the shares of the total population for various contributor categories from 2020 to 2022.

The share of wage workers increased by 2.3%, which may reflect an increase in the number of job opportunities or changes in the economy (*t* = 162.6, *p* ≤ 0.001). Individual entrepreneurs and those engaged in private practice showed an increase in their share of the total population by 0.4%, indicating an increase in the number of entrepreneurs or the development of private practices (*t* = 94.3, *p* ≤ 0.001). The share of self-employed payers significantly increased over the same period, indicating growth in the number of self-employed individuals (*t* = 188.7, *p* ≤ 0.001). Individuals entitled to receive medical assistance under the mandatory social medical insurance system saw their share increase by 1.5%, which may be related to the expansion of medical insurance coverage (*t* = 117.1, *p* ≤ 0.001).

Conversely, the share of individuals for whom contributions and payments were made decreased by 5.6% from 2020 to 2022, which may indicate changes in the social security system or changes in labor relations (*t* = 396.0, *p* ≤ 0.001). The share of individuals exempt from contributions decreased by 0.9% over the same period, potentially related to changes in social insurance legislation (*t* = 63.6, *p* ≤ 0.001). The share of individuals working under civil law contracts remained roughly the same over the 3 years (*t* = 0.00, *p* ≥ 0.05). Unified aggregate payment payers showed a significant reduction in their share of the total population from 8.8 to 1.0%, possibly due to changes in legislation or working conditions (*t* = 1071.4, *p* ≤ 0.001). The share of uninsured individuals slightly decreased by 0.5% over the same period (*t* = 117.1, *p* ≤ 0.001).

This comparative analysis helps identify key trends in the changing shares of various contributor categories relative to the total population and indicates possible changes in the socio-economic sphere.

Overall, the execution of the plan for the Guaranteed Volume of Free Medical Care is 100.0%, and for the CSHI on average 99.95%. The difference between the percentages is insignificant and may be due to differences in budgetary constraints, funding procedures, or other factors. The overall execution of the plan for all types of medical care is 100.0%, indicating sufficient financial support for the healthcare system. Small differences in percentages between Guaranteed volume of free medical care and CSHI may require additional analysis to determine the reasons and take appropriate measures (Table 3).

**Table 3:** Execution of the procurement plan by type of medical services in 2022.

Types of medical care	Execution	GVFMC	CSHI	GVFMC, %	CSHI,%
Program dialysis	42,292,345	42,292,345		100.0%	
High-tech medical services	67,766,175	12,211,113	55,555 062	100.0%	100.0%
The complex consultative-diagnostic unit (CCDU)	277,592,440	36,303,209	241,289,231	100.0%	99.84%
HIV AIDS	20,080,737	20,080,737		100.0%	
Infection	59,569,484	59,569,484		100.0%	
Psychiatry, drug addiction, alcoholism	46,570,411	46,570,411		100.0%	
Tuberculosis	45,168,360	45,168,360		100.0%	
Oncohematology	25,414,481	21,748,863	3,665,618	100.0%	100.0%
Oncology	95,091,824	95,091,824		100.0%	
Payment for COVID 19 in the Republic of Kazakhstan	6,837,440		6,837,440		99.77%
Palliative care	5,360,110	5,360,110		100.0%	
Pathological diagnosis	1,534,019	789,738	744,280	100.0%	100.0%
Primary health care	449,629,373	449,629,373		100.0%	
Ambulance and air ambulance	76,805,998	76,805,998		100.0%	
Village hospital	131,270,086	49,449,118	81,820,968	100.0%	100.0%
Inpatient medical care	431,822,989	87,071,287	344,751,702	100.0%	100.0%
Inpatient medical care	65,719,584	24,815,412	40,904,171	100.0%	99.94%
Blood services	27,426,787	27,426,787		100.0%	
Medical rehabilitation	60,405,584		60,405,584	100.0%	
Total	1,936,358,233	1,100,384,174	835,974,059	100.0%	99.95%

Table 4 presents data on waiting times for consultative-diagnostic services across various healthcare sectors, including rehabilitation centers, pathological diagnostics, oncological disease diagnosis, COVID-19 testing, and others. The data shows the number of services referred and waiting times categorized into intervals from 0 to 3 days up to over 30 days.

**Table 4:** Waiting times for consultative and diagnostic services of outpatient care in the city of Almaty.

Type of service	Number of areas of advisory and diagnostic services outside the comprehensive per capita standard	Total expected	Of which they expect:				
			0–3 days	4–10 days	11–20 days	21–30 days	More than 30 days
Complex of advisory and diagnostic services for 1 assigned resident	8,214,946 (90.1 ± 0.01)	6,919 (80.3 ± 0.43)	3,916 (91.7 ± 0.4)	2,185 (76.6 ± 0.79)	652 (74.6 ± 1.47)	95 (49.2 ± 3.6)	71 (16.6 ± 1.8)
Services in rehabilitation centers	383,598 (4.21 ± 0.007)	296 (3.43 ± 0.20)	141 (3.30 ± 0.27)	133 (4.66 ± 0.39)	7 (0.80 ± 0.30)		15 (3.50 ± 0.9)
Pathological diagnostics aimed at intravital diagnosis of diseases (cytological and histological studies)	3,262 (0.04 ± 0.001)	915 (10.6 ± 0.33)	127 (29.7 ± 0.26)	459 (16.1 ± 0.69)	146 (16.7 ± 1.26)	24 (12.4 ± 2.4)	159 (37.1 ± 2.3)
Services of specialized medical care in an outpatient setting for the diagnosis of neoplasms, dynamic monitoring of cancer patients	91,722 (1.01 ± 0.003)	155 (1.80 ± 0.14)	70 (1.64 ± 0.19)	43 (1.51 ± 0.23)	23 (2.63 ± 0.54)	14 (7.3 ± 1.9)	5 (1.17 ± 0.5)
COVID-19 screening services	130,967 (1.44 ± 0.004)						
Pathological autopsy (autopsy)	74 (0.001 ± 0.00)	30 (0.35 ± 0.06)		1 (0.04 ± 0.04)	7 (0.80 ± 0.30)	7 (3.6 ± 1.3)	15 (3.50 ± 0.9)
Services in dermatovenerological clinics (and/or departments at multidisciplinary hospitals)	192,933 (2.12 ± 0.005)						
High-tech medical services guaranteed volume of medical care	11,581 (0.13 ± 0.001)	292 (3.39 ± 0.19)	11 (0.26 ± 0.08)	29 (1.02 ± 0.19)	38 (4.35 ± 0.69)	53 (27.5 ± 3.2)	161 (37.6 ± 2.3)
Services at youth health centers	14,479 (0.16 ± 0.001)						
Medical rehabilitation in outpatient clinic care for coronavirus infection COVID-19	1,420 (0.02 ± 0.00)						
Expensive types of diagnostic tests for patients with suspected cancer upon referral from a specialist	6,722 (0.07 ± 0.001)	5 (0.06 ± 0.03)		2 (0.07 ± 0.05)	1 (0.11 ± 0.11)		2 (0.47 ± 0.3)
Services for pensioners and family members of military personnel, law enforcement and specialized government agencies	5,800 (0.06 ± 0.001)	5 (0.06 ± 0.03)	5 (0.12 ± 0.05)				
Expensive types of diagnostic tests for patients with suspected cancer on the referral of a specialist: Computed tomography, Magnetic resonance imaging	4,289 (0.05 ± 0.001)	1 (0.01 ± 0.01)		1 (0.04 ± 0.04)			
Preventive medical examinations of target population groups	49,968 (0.55 ± 0.002)						
Medical care for schoolchildren in educational organizations	4,238 (0.05 ± 0.001)						

Rehabilitation centers exhibit the shortest average waiting time (4.21 ± 0.007), indicating good availability of these services. Pathological diagnostics also demonstrate a low average waiting time (0.04 ± 0.001), suggesting rapid availability. In contrast, comprehensive consultative-diagnostic services show a significantly higher average waiting time (90.1 ± 0.01), potentially indicating limited availability or higher workload. COVID-19 testing services lack a specified average waiting time, suggesting prompt processing and provision.

This analysis helps evaluate the effectiveness and accessibility of different healthcare sectors and identifies potential organizational issues. Notably, rehabilitation centers and COVID-19 testing services stand out for their shorter waiting times, while comprehensive consultative-diagnostic services require substantially longer waiting periods.

Table 5 presents an analysis evaluating the differences between Guaranteed Volume of Free Medical Care (GVFMC) and Compulsory Social Health Insurance (CSHI) in providing consultative-diagnostic services, including the number of services rendered, waiting times, and payment costs. The Complex Consultative-Diagnostic Unit (CCDU) per capita attachment is as follows: GVFMC: 8263177 services rendered with an average waiting time of 90.0 days, billed for 19,197,328,523 tenge. CSHI: 1231255 services rendered with an average waiting time of 82.7 days, billed for 3,063,363,573 tenge. Both types of services have a significant number of billed services, with the waiting time slightly higher for GVFMC than for CSHI. Services in rehabilitation centers are as follows: GVFMC: 383916 services rendered with an average waiting time of 4.18 days, billed for 484,023,130 tenge. CSHI: Data unavailable. GVFMC provided a greater number of services in this area with a lower waiting time. Pathological diagnostics: GVFMC: 11799 services rendered with an average waiting time of 0.13 days, billed for 78,560,224 tenge. CSHI: 3447 services rendered with an average waiting time of 0.23 days, billed for 22,976,809 tenge. Both types of services have low waiting times, but GVFMC provided a greater number of services.

**Table 5:** Consultative and diagnostic services of outpatient care within the framework of the statewide volume of medical care and compulsory medical insurance.

Type of service	Number of areas of advisory and diagnostic services outside the comprehensive per capita standard	Number of services				Presented for payment, in tenge	Presented for payment	
		GVFMC	CSHI	Paid for the uninsured	Other		GVFMC	CSHI
Complex of advisory and diagnostic services for 1 assigned resident	8,263,177 (90.0 ± 0.10)	1,231,255 (82.7 ± 0.03)	7,030,294 (91.4 ± 0.010)	1,447	181	19,197,328,523	3,063,363,573	16,133,964,951
Services in rehabilitation centers	383,916 (4.18 ± 0.007)		383,916 (4.99 ± 0.008)			484,023,130		484,023,130
Pathological diagnostics aimed at intravital diagnosis of diseases (cytological and histological studies)	11,799 (0.13 ± 0.001)	3,447 (0.23 ± 0.004)	8,352 (0.11 ± 0.001)			78,560,224	22,976,809	55,583,415
Services of specialized medical care in an outpatient setting for the diagnosis of neoplasms, dynamic monitoring of cancer patients	101,981 (1.11 ± 0.003)	101,018 (6.79 ± 0.02)	963 (0.01 ± 0.004)			1,068,902,453	1,064,229,127	4,673,326
COVID-19 screening services	130,973 (1.43 ± 0.004)	130,973 (8.80 ± 0.02)				621,903,497	621,903,497	
Pathological autopsy (autopsy)	75 (0.001 ± 0.0001)	15 (0.001 ± 0.0003)	60 (0.001 ± 0.0001)			8,173,543	1,685,029	6,488,513
Services in dermatovenerological clinics (and/or departments at multidisciplinary hospitals)	192,935 (2.10 ± 0.005)	3,333 (0.22 ± 0.004)	189,602 (2.46 ± 0.006)			390,574,157	6,953,283	383,620,874
High-tech medical services guaranteed volume of medical care	11,581 (0.13 ± 0.001)	11,581 (0.78 ± 0.01)				3,331,049,168	3,331,049,168	
Services at youth health centers	14,479 (0.16 ± 0.001)		14,479 (0.19 ± 0.002)			33,177,071		33,177,071
Medical rehabilitation in Outpatient clinic care for coronavirus infection COVID-19	1,429 (0.02 ± 0.0004)		1,429 (0.02 ± 0.0005)			2,486,035		2,486,035
Expensive types of diagnostic tests for patients with suspected cancer upon referral from a specialist	6,722 (0.07 ± 0.001)	6,722 (0.45 ± 0.01)				1,805,261,530	1,805,261,530	
Services for pensioners and family members of military personnel, law enforcement and specialized government agencies	5,800 (0.06 ± 0.001)	452 (0.03 ± 0.001)	5,348 (0.07 ± 0.001)			15,250,318	1,160,360	14,089,959
Expensive types of diagnostic tests for patients with suspected cancer on the referral of a specialist: Computed tomography, Magnetic resonance imaging	4,289 (0.05 ± 0.001)		4,289 (0.06 ± 0.001)			117,468,638		106,704,386
Preventive medical examinations of target population groups	49,968 (0.54 ± 0.002)		49,968 (0.65 ± 0.003)			119,370,530		119,370,530
Medical care for schoolchildren in educational organizations	4,238 (0.05 ± 0.001)		4,238 (0.06 ± 0.001)			10,431,879		10,431,879

Specialized medical services for diagnosing neoplasms: GVFMC: 101,981 services rendered with an average waiting time of 1.11 days, billed for 1,068,902,453 tenge. CSHI: 101018 services rendered with an average waiting time of 6.79 days, billed for 1,064,229,127 tenge. GVFMC has a lower waiting time and provided a greater number of services compared to CSHI. Services for COVID-19 testing: GVFMC and CSHI: 130973 services rendered with average waiting times of 1.43 and 8.80 days, respectively, billed for 621,903,497 tenge. The waiting time for GVFMC is significantly lower than that for CSHI.

## 4 Discussion

The research results have identified differences in demand for medical services depending on the type of insurance, with personal insurance being the most profitable. Policy-makers should leverage this behavioral response by introducing insurance packages that share insurance contributions with citizens to incentivize their utilization (22). Although insurance systems (both individual and social) are considered a more equitable form of financing, direct payment for medical services is viewed as less equitable. In many countries, there are still issues with various payment methods, and people resort to direct payments (23).

Healthcare managers identify delays and uneven reimbursement of expenses as the main problem. It is proposed to seek alternative sources of funding, including special taxes on natural resources and an increase in value-added tax (VAT) (24). One of the most significant problems faced by insurance companies is a high loss ratio (25).

In Central and Eastern European countries, it is necessary to strengthen the role of state financing by prioritizing budget allocations, reducing the share of out-of-pocket payments for medical goods and medications, expanding access to subsidized service packages, and improving the quality of healthcare (26).

Healthcare financing and achieving sustainable development goals by 2030 have become priorities for many low- and middle-income countries. Improving public healthcare and reducing personal medical expenses are key goals of healthcare policy in the Asia-Pacific region (27).

To ensure fairness in healthcare financing, decisive steps need to be taken to reduce inequality in funding. This can be achieved by transitioning to contributions adapted to financial capabilities, increasing the role of tax financing, and reducing reliance on out-of-pocket payments and private health insurance (28).

A healthcare policy based on a universal health insurance system should aim to expand insurance coverage for chronic recurring diseases, which pose financial challenges and require special attention in financing strategies (29). The study highlights the paradox of social responsibility in providing quality healthcare services and professional autonomy, which may be affected by the healthcare financing structure, negatively impacting incomes and workload. Efforts to reform healthcare financing and delivery in the United States may encounter resistance from healthcare service providers if these reforms impinge on their professional autonomy (30).

The limitations of this study include the lack of data on the workforce structure across economic sectors and regions. There is insufficient information regarding the quality of healthcare services provided and their impact on population health. Considering these assumptions and limitations will help form a more comprehensive and accurate understanding of the workforce and healthcare status, as well as identify directions for further research.

The groundwork for future research could include studying the impact of demographic changes on shifts in the workforce composition and unemployment rates. Analyzing the factors influencing entrepreneurial activity and the creation of new jobs. Investigating the reasons for the rise in long-term unemployment and the increase in the average duration of unemployment. Assessing the effectiveness of measures aimed at reducing waiting times and increasing the volume of services provided within both healthcare systems.

Policy implications: This study highlights key differences in the demand for medical services between Compulsory Social Health Insurance (CSHI) and the Guaranteed Volume of Free Medical Care (GVFMC) in Kazakhstan. The findings suggest that policy-makers should consider introducing insurance packages that share contributions with citizens to encourage greater utilization of services. Additionally, reducing reliance on direct payments by improving insurance coverage could enhance equity in healthcare financing.

To address challenges like delays and uneven reimbursement, exploring alternative funding sources such as special taxes on natural resources and increasing VAT could stabilize healthcare financing. Strengthening state financing, reducing out-of-pocket payments, and improving access to subsidized services are essential steps for improving healthcare quality and equity.

Innovation and contributions: The study provides new insights into the accessibility and quality of services under CSHI and GVFMC, offering a unique perspective on optimizing healthcare delivery in Kazakhstan. It also highlights the tension between healthcare financing reforms and the professional autonomy of service providers, a crucial consideration for policy-makers.

Limitations and future research: The study is limited by the lack of detailed data on workforce structure and the quality of healthcare services. Future research should explore the impact of demographic changes on employment, the factors driving entrepreneurial activity, and the effectiveness of strategies to reduce waiting times and increase service volumes.

## 5 Conclusion

In the Compulsory Social Health Insurance (CSHI), there has been an increase in the number of insured individuals, especially among privileged groups and wage workers. However, some contributors, including those exempt from contributions and the Unified Social Payment (USP), are reducing their share, possibly due to changes in legislation or the economy. The Guaranteed Volume of Free Medical Care (GVFMC) offers more services with shorter waiting times but higher costs, indicating potentially more efficient medical services under the state program.

## Data Availability

The original contributions presented in the study are included in the article/supplementary material, further inquiries can be directed to the corresponding author.
